# Society Meetings

**Published:** 1909-12

**Authors:** 


### SOCIETY MEETINGS.

THE American Academy of Medicine held its third mid-year meeting (specialising in medical sociology), at New Haven, November 11 and 12, 1909. The meeting was principally a conference on prevention of infant mortality. A number of papers were read and discussed, and at the close of the conference, the American Association for Study and Prevention of Infant Mortality was organised at a special meeting presided over by Dr. Frederick H. Gerrish, of Portland, Me.

---

THE Buffalo Academy of Medicine held meetings during the month of November, 1909, as follows:

*Section of Surgery.*—Wednesday evening, November 3. Program: A lantern slide demonstration of some diseases of the skin, Wm. Allen Pusey, Chicago, Ill.

*Section of Medicine.*—Tuesday evening, November 9. Program: (a) Early treatment of the insane; and proposed new legislation, Arthur W. Hurd; (b) Psychopathic hospitals, James W. Putnam. Discussion opened by D. H. Arthur, superintendent of Gowanda State Hospital.

*Section of Pathology.*—Tuesday, November 16. Program: The nurse in tuberculosis, Mabel Jacques; Some aspects of serum diagnosis of syphilis, J. G. Fitzgerald.

*Section on Obstetrics and Gynecology.*—Tuesday, November 23. Program: E. E. Montgomery, M.D., L.L.D., Philadelphia, Pa., Professor of Gynecology, in the Jefferson Medical College; Gynecologist to the Jefferson and St. Joseph's Hospitals. Subject: The significance and treatment of hemorrhage from the genital tract. Discussion opened by M. D. Mann.

---

THE Rochester Academy of Medicine held meetings during the month of November, 1909, at the Hotel Seneca, as follows:

*Public Health.*—Wednesday, November 3. Program: Preliminary report on the tuberculin test as applied to a city's milk supply. Illustrated with specimens, George W. Goler.

*General Medicine.*—Wednesday, November 17. Program: Some cases treated by vaccines, C. O. Boswell.

*Obstetrics, Gynecology, and Pediatrics.*—Wednesday, November 24. Program: Pregnancy as a casual factor in enteroptosis, Richard Moore.

